# Health care waste management in community-based care: experiences of community health workers in low resource communities in South Africa

**DOI:** 10.1186/s12889-017-4378-5

**Published:** 2017-05-15

**Authors:** Lydia Hangulu, Olagoke Akintola

**Affiliations:** 1 Health Promotion Postdoctoral Programme, Discipline of Psychology, University of KwaZulu-Natal, MTB Ground Floor, 1X09, Durban, 4041 South Africa; 2 Health Promotion Programme, Discipline of Psychology, University of KwaZulu-Natal, 4041, King George Avenue, Durban, 4041 KwaZulu-Natal South Africa

**Keywords:** Community-based care, Community health workers, Health care waste, HIV/AIDS

## Abstract

**Background:**

In South Africa, community health workers (CHWs) working in community-based care (CBC) programmes provide care to patients most of whom are living with HIV/AIDS and tuberculosis (TB). Although studies have shown that the caregiving activities provided by the CHWs generate health care waste (HCW), there is limited information about the experiences of CHWs on health care waste management (HCWM) in CBC. This study explored HCWM in CBC in Durban, South Africa from the perspectives CHWs.

**Methods:**

We used three ethnographic approaches to collect data: focus group discussions, participant observations and informal discussions. Data was collected from 85 CHWs working in 29 communities in the Durban metropolis, South Africa. Data collection took place from July 2013 to August 2014.

**Results:**

CHWs provided nursing care activities to patients many of whom were incontinent or bedridden. Some the patients were living with HIV/AIDS/TB, stroke, diabetes, asthma, arthritis and high blood pressure. These caregiving activities generate sharps and infectious waste but CHWs and family members did not segregate HCW according to the risk posed as stipulated by the HCWM policy. In addition, HCW was left with domestic waste. Major barriers to proper HCWM identified by CHWs include, lack of assistance from family members in assisting patients to use the toilet or change diapers and removing HCW from homes, irregular waste collection by waste collectors, inadequate water for practicing hygiene and sanitation, long distance between the house and the toilets and poor conditions of communal toilets and pit latrines. As a result of these barriers, HCW was illegally dumped along roads or in the bush, burnt openly and buried within the yards. Liquid HCW such as vomit, urine and sputum were disposed in open spaces near the homes.

**Conclusion:**

Current policies on primary health care (PHC) and HCWM in South Africa have not paid attention to HCWM. Findings suggest the need for primary health care reform to develop the competencies of CHWs in HCWM. In addition, PHC and HCWM policies should address the infrastructure deficit in low resource communities. In order for low-and-middle-income-countries (LMICs) to develop effective community health worker programmes, there is a need for synergies in PHC and HCWM policies.

## Background

Globally, the management of health care waste poses a major environmental and public health challenge [1]. Health care waste (HCW) is all waste that is generated in health care facilities such as hospitals, clinics, pharmaceutical manufacturing plants, research laboratories, nursing homes and other settings like homes where there is care for a patient [2, 3]. In low-and-middle-income-countries (LMICs), the management of HCW is particularly challenging [4, 5]. For example, in most African countries, there is insufficient knowledge on how to handle HCW among community health workers (CHWs) and other staff working in health care settings [6]. Moreover, HCW is often not properly segregated from point of generation to the point of disposal and most dumpsites are poorly managed with scavengers gaining access to the dumpsites [7]. Studies also reveal that there is limited financial investment and a lack of clear policies to manage HCW in many LMICs [8, 9]. This together with inadequate technologies for managing HCW, makes incineration the most common method for disposing HCW [10].

Improper health care waste management (HCWM) has negative ramifications for a variety of people, including exposing the general public, health care workers, waste handlers, caregivers, patients, waste pickers and animals to injuries [1, 2]. In India, a study on HCWM revealed that more than 30% of the 3–6 billion injections that were administered every year, in almost 10 of the health care facilities nationwide, were done with used syringes recycled by unskilled scavengers who sold them on the black market [11]. Furthermore, CHWs, waste handlers and the general public who are exposed to HCW could be at risk of infection with hepatitis A, B and C [1, 12]. Exposure to HCW can cause diseases like diarrhoea, leptospirosis, typhoid, cholera, and HIV and tuberculosis (TB) all of which could cause death [2, 13]. Indeed, approximately 5.2 million people globally die every year due to diseases caused by improper management of HCW [14].

In South Africa, as in many other African countries, HCW is improperly managed. A desktop study in KwaZulu-Natal found that about 45% of HCW in the province is unaccounted for [15]. Another study conducted on HCWM in 30 health care clinics in a rural health district in KwaZulu-Natal province found that, HCW was not segregated from point of generation in all the facilities. In addition, four of the 30 facilities burnt and buried HCW in shallow pits within the premises of the clinics [16].

While researchers have underscored the challenges associated with HCWM in healthcare facilities, the waste that emanates from community-based care (CBC) settings should be of greater concern because unlike in hospitals, homes are not built to accommodate HCW [17, 18]. Yet, there is very limited information about HCWM in CBC. In South Africa, community-based organisations (CBOs) are heavily relied upon to provide health and social services to poor and marginalised communities [19]. The CBOs provide on-going care to people with illnesses such as HIV/AIDS, TB and cancer in their homes with the help of CHWs living in these communities [20]. Research has shown that the care-related activities provided by CHWs could potentially generate HCW [21–24]. However, issues relating to HCWM in CBC have rarely been explored in the literature.

At the same time, current reforms in primary health care (PHC) in South Africa seek to scale up CBC through the re-engineering of PHC, a fundamental component of the national health insurance (NHI) [25]. The PHC reform which has been rolled out in pilot sites since 2012 entails the use of outreach teams led by nurses providing care in marginalised communities across the country [25]. The scaling up of health care services provision in CBC brings into sharp focus the issue of HCWM in homes and communities. Given the potential for CBC activities to generate HCW [21], and the primary health care policy reforms that seeks to promote CBC on a national scale [26], there is the need to understand the management of HCW in homes where care activities are provided. However, we found no study exploring the perspectives of CHWs about HCWM. This study therefore aims to explore how HCW is managed in CBC from the perspective of CHWs. The findings could help inform environmental as well as public health policy and practices in South Africa.

## Methods

We chose to use a qualitative study design because we found it appropriate in exploring the subjective perceptions and experiences of participants within their social context [27–29]. The qualitative approach allowed us to explore the experiences of CHWs in relation to HCWM in CBC in their own language.

### Study setting and context

We conducted the study in KwaZulu-Natal (KZN), the province with the highest HIV prevalence rates (40%) [30, 31] and 76% of the people living with HIV/AIDS (PLWHA) are also co-infected with TB [32]. We recruited participants from thirteen CBOs providing health and social services in 29 low resource communities located on the outskirts of Durban, South Africa. These organsations were founded between 1996 and 2006 and provided social services in various communities. The communities comprised of three informal settlements, 21 peri-urban and five rural communities. Informal settlements are defined as communities that are occupied by migrants from rural areas or foreign nationals who are in search of employment and social services [33]. Municipal services in these areas are often free, very basic but are mostly unavailable [32]. Peri-urban communities are neither rural nor urban; they are densely populated and underdeveloped [33]. Rural communities mainly consist of scattered elderly populations who rely on social grants. Municipal services in these areas are also basic and free. All these communities are occupied by poor people (indigenous African people classified as blacks under the apartheid era) [33] and they all fall within the jurisdiction of eThekwini metropolitan municipality (see Table 1 for demographic information).

**Table 1:** Demographics of the organisations

Name of CBOs	Location of the organisations	Year the organisation was founded	Number of communities served	Number of participants in FGDs
A	Informal settlement	2006	3	8
B	Rural area	1999	2	7
C	Peri-urban	1998	2	6
D	Peri-urban	2001	3	7
E	Peri-urban	1992	2	7
F	Peri-urban	2002	1	6
G	Peri-urban	1999	2	5
H	Peri-urban	2000	2	8
I	Peri-urban	2000	1	8
J	Peri-urban	1990	3	6
K	Peri-urban	1996	2	6
L	Peri-urban	1999	3	5
M	Peri-urban	1997	3	6

All organisations provided services related to HIV/AIDS/TB, drug abuse, rape and antiretroviral treatment (ART). They offered life skill programmes such as gardening and bead work. Basic counselling programmes were also offered to patients to help them to accept their HIV positive status, develop coping mechanisms such as dealing with stigma, adherence to ART and coping with bereavement. Social services offered include referrals to relevant social support structures, help with accessing pension grants for the elderly citizens, child grants and with processing national identity cards. Furthermore, these organisations provided CBC and day care services for orphans and vulnerable children. Activities carried out in CBC are basic nursing care offered to PLWHA, TB and to those who have chronic illnesses such as diabetes and hypertension.

### Participants

We used snowball sampling, a technique used to locate participants through referrals [34] to identify CBOs providing CBC. At the outset, we approached two organisations that we had worked with previously who in turn provided referrals to other organisations that they had partnered with. All organisations were selected purposively if they were willing to participate in the study and if they provided CBC to PLWHA, TB and other chronic illnesses. This was because we believed that CHWs working in such organisations were in a better position to provide insights into HCWM. In order to include participants with deep insights into the research question [35], we recruited participants only if they met the following criteria: 1) if they worked for any of the CBOs providing CBC to PLWHA, TB, diabetes, hypertension and other illnesses as discussed earlier, and 2) if they had at least 6 months of work experience. A total of 85 CHWs who met the inclusion criteria were recruited to participate in the study. All participants were females and their ages ranged between 23 and 60. Their work experience ranged between one and 21 years (see Table 2).

**Table 2:** Characteristics of community health workers

Name of the organisations	Number of participants in FGDs	Range of Years of service in CBOs	Age range
A	8	2–6	23–39
B	7	1–2	23–39
C	6	3–9	34–59
D	7	2–15	27–54
E	7	1–6	29–48
F	6	2–10	34–54
G	5	2–19	38–45
H	8	1–10	32–44
I	8	1–2	27–52
J	6	1–8	33–37
K	6	6–21	30–50
L	5	1–8	37–60
M	6	2–3	31–41
Total:13	85	Mean (μ) = 5.3	Mean (μ) = 38.8

### Data collection procedure

We employed three ethnographic research approaches to collect data: focus group discussions, participant observations and informal discussions. These approaches were appropriate for triangulation purposes which aim at eliminating the researchers’ biases, and improving the quality of data [36]. Firstly, we conducted focus group discussions (FGDs), in order to capture a wide range of perspectives and to understand normative practices [37] about HCWM. In all, we conducted 13 FGDs, one per organisation with about 5 to 8 CHWs in each FGD (Table 2). All FGDs were guided by a focus group schedule which contained open-ended questions. The schedule provided participants with the opportunity to provide detailed responses and for the facilitator to probe and follow-up on issues raised during the sessions [33]. The focus group schedule covered four main themes: 1) nursing care activities provided to the patients and the type of HCW that is generated in the process, 2) how HCW is handled and managed in CBC, 3) barriers and facilitators to the management of HCW and 4) strategies used to respond to challenges relating to HCWM. The FGDs were conducted in meeting rooms provided by the organisations. All FGDs were conducted in IsiZulu, the main local language spoken in the communities and were facilitated by a trained research assistant who was a native IsiZulu speaker. The FGDs lasted between 60 and 85 min. Data collection took place from July 2013 to August 2014.

Secondly, following all FGDs, observations were conducted on the HCWM practices of CHWs within the homes of the patients to improve data quality and interpretation [38]. The CHWs were accompanied to the homes of the patients where they conducted their daily care work. Permission was also obtained from families to conduct observations of the state of the homes and HCWM practices. At the household level, the environment of all households was observed and documented. These included the availability, type and state of toilets in the households, access to water facilities, waste storage facilities and equipment such as house bins and garbage bags. In each home, we also observed the hygiene and HCWM practices of the CHWs. At the community level, we observed the location of communal toilets and their distance to the patients’ homes, accessibility of roads, community waste storage facilities and the general cleanliness of the environment (streets, paths, cliffs, surrounding bushes and some streams where necessary). Thirdly, during observations, informal discussions were conducted with CHWs to clarify issues relating to HCWM practices. The observed activities, events and responses provided from the informal discussions were written down in a note book.

### Data analysis

All the recorded data from FGDs was transcribed verbatim and translated from IsiZulu into English by the research assistant who facilitated the FGDs. Both authors met to discuss how to apply the six steps of thematic analysis [39]. One of the authors [LH] conducted the analysis in active discussion with the other author [OA]. Both authors met regularly to review the coding framework. We followed the six steps proposed by Braun and Clarke [39]: 1) in order to familiarise ourselves with the data, we read all the transcripts from the focus group discussions, field notes obtained during observations and informal discussions; 2) we read the data to identify and generate the codes; 3) we identified themes from all the codes that we generated; 4) we reviewed all identified themes and developed sub-themes; (5) all the reviewed themes and sub-themes were grouped together, and 6) all the grouped themes are presented in the results.

## Results

The findings are presented under three broad themes identified from the data. The first theme describes the nature of nursing care activities and how HCW is generated in CBC. The second describes how HCW is handled and managed in homes of the patients. The third theme describes the barriers to the management of HCW and the strategies that CHWs use to deal with the barriers. All themes are discussed together with sub-themes and are supported by direct quotes from the participants.

### Activities responsible for generating health care waste in community-based care

In order to understand the activities that contribute to generating HCW in CBC, we asked CHWs participating in the focus group sessions about the services that they provide to the patients on a day-to-day basis and the waste that is generated in the process. CHWs’ responses revealed that they provide nursing care activities to HIV/AIDS patients who are incontinent, or bedridden. They also provide care to patients who have stroke or are diabetic. Some of the diabetic patients have wounds resulting from amputations while HIV patients have opportunistic infections such as TB and diarrhoea. Many of the patients also have other diseases such as asthma, arthritis and high blood pressure.

CHWs provide nursing care activities such as cleaning and dressing of wounds, changing of diapers, bed baths, brushing of teeth, washing of linen and administering insulin injections. These nursing care activities generate waste materials such as used gloves that CHWs wear when providing care to the patients, soiled diapers, swabs, used bandages and soiled linen. These materials are typically contaminated with faecal matter and bodily fluids such as urine, blood, vomit, pus, sputum and phlegm. There is also waste water resulting from giving bed baths to the patient and also from cleaning containers used to contain sputum, phlegm and vomit from patients with nausea and TB. CHWs described instances where incontinent patients and those that are too weak to walk to the toilet use buckets for toileting purposes because they cannot afford to buy diapers.

*“Some patients cannot walk to the toilet. They pee (urinate) and do everything on themselves, so at the end of the day you find that we have urine…even faeces in a bucket because some patients do not have diapers so you put a bucket for them to use”* (Focus Group D).

### Health care waste management practices in community-based care

CHWs indicated that they received training that equips them with knowledge and skills on how to handle HCW. They have the knowledge that HCW is hazardous and that they must wear gloves when handling it in order to protect themselves from infections. In the absence of gloves they improvise by wearing plastic bags. CHWs pass on this knowledge to the family members of the patients by training them. During FGDs, CHWs said that they apply the skills that they learnt during training in practice. This position is consistent with what we observed during our home visits. We saw CHWs wearing gloves or plastic bags when handling HCW. We also noted that they seemed to be disgusted when handling HCW due to the fact that it has a repulsive smell.

However, contrary to their training, some participants indicated that, there are occasions when they do not wear plastic bags as a substitute for gloves because it is difficult to manipulate all their fingers when using plastic bags. Some participants cited instances when they wound up handling HCW with their bare hands despite wearing plastic bags. We confirmed this assertion during observations. In addition, we observed some CHWs changing diapers of the patients, picking up all the used diapers and the containers containing vomit, sputum and phlegm with their bare hands. When we asked why they did this during informal discussions, one of the CHWs said that she was pressed for time to attend to other patients and that she would wash her hands afterwards to prevent any infections.

Although CHWs indicated that they received training regarding segregation of HCW during FGDs, observations revealed that the training only equipped them with knowledge and skills to segregate HCW from domestic waste as such, within the homes, CHWs did not separate HCW according to the risk it posed. In addition, none of the households that we visited were provided with yellow containers for storing sharps, and as a result, all sharp waste was mixed with other HCW. We observed that neither the CHWs nor the households were provided with red plastic bags to store infectious waste as stipulated by the South African Standards [40]. As a consequence, all households and CHWs stored all HCW in black plastic bags meant for domestic waste. CHWs blamed the municipality for not supplying red plastics for storing HCW. They also blamed the lack of gloves on the government agencies responsible for CBC as well as international and local donor agencies.

During FGDs, participants indicated that prior to the year 2010, most CBC programmes were funded by the European Union and that CBOs had fairly regular supply of CBC kits from the Department of Health which contained all necessary supplies for providing nursing care. According to them, the kits contained items such as bandages, swabs, sanitizers, gloves, masks, medication, linen savers and aprons and red plastic bags. Participants indicated that they used to store all HCW in the red plastics and thereafter, took the red plastics containing HCW to their organisations from where the HCW was transported to local clinics and finally taken for disposal by the relevant authorities. However, they noted that there has been a shortage of CBC kits since the year 2010 when most CBOs experienced funding cuts.

At the time of the study, none of the CBOs were receiving any kits or materials from the Department of Health. CHWs indicated that they have been relying on alternative sources of donations such as the nearest clinics, individual donors and local business owners for these materials. CHWs explained that they take extra precautions in handling the HCW that they generate but that they store them in house bins together with domestic waste.

“…*We put everything in a big plastic [black plastics] and tie it [them] so that children will not access it [them] because they like to get gloves to use as balloons…”* (Focus Group D).

In informal settlements, most houses are clustered together and as a result, there are no formal roads. The municipality provides communal waste storage facilities that are located close to the main roads and all households are expected to store their waste in these facilities on a daily basis. On the other hand in peri-urban and rural communities, where there are roads, all households are expected to have bins for storing their waste and to remove the waste to the kerbside for collection by the waste collectors on particular waste collection days. Observations on environmental hygiene in homes showed that some households in the informal settlements and peri-urban communities did not have house bins. In such homes, black garbage bags containing HCW were stored in a hidden corner outside the houses without proper protection or supervision, making it accessible to children who were seen scavenging for toys.

### Barriers to proper health care waste management

CHWs identified the following barriers to the proper management of HCW in homes: lack of co-operation from family members, irregular waste collection services by waste collectors, inadequate water to practice hygiene and sanitation, and long distances between the toilets and the homes of the patients.

#### Lack of assistance from family members

CHWs indicated that patients who are too weak to walk use buckets for toileting while patients with TB use tin containers to vomit and spit because of a shortage of material supply and the fact that the families are unable to afford these materials. In the absence of CHWs, family members are supposed to help lift the incontinent and weak patients so they could use the buckets. They are also expected to help empty and clean all buckets for collecting HCW, change diapers and remove all the HCW that is generated in homes to the kerbside for collection. CHWs reported, however, that most family members did not provide the needed care to incontinent and weak patients. As a consequence, some of the patients end up relieving themselves on their beds thereby developing bedsores. In many instances, family members did not remove HCW from the rooms. This made the patients’ rooms and indeed some homes to have a repulsive smell.

*“… They do not change the diapers, they do not dispose of the diapers that we leave behind, instead we find them in the same room where the patient is when we come back. So how can a person heal like that? It is just not fair! You find that the room is smelling because of the nappies [soiled diapers]…”* (Focus Group J).

With such a challenge, CHWs expressed feeling helpless and frustrated yet they continue to educate the family members about HIV/AIDS and the importance of practicing hygiene to promote the wellbeing of the patient.

*“There is nothing (else) we can do…but we teach the family members how to take care of a patient because that is what we are trained to do and we cannot force them to do it if they do not want to…”* (Focus Group A).

#### Irregular waste collection by waste collectors

All households in the communities are provided with waste collection schedules and are expected to remove all waste from homes to the kerbside once a week on a particular day set for waste collection. However, CHWs indicated that waste was left uncollected on several occasions. CHWs and households said that they are ‘left in the dark,’ which means that the waste collectors provide them with no warnings or reasons for not collecting waste. Consistent with findings from FGDs, we observed that waste was usually left on the kerbsides for long periods of time, and was blown away by strong winds and scattered by dogs.

*“We put the plastic bags on the road side every Wednesday so that [the] municipality will pick it up but you find that they do not come so it is blown away by [the] wind…also dogs come and tear it up and the waste will be all over everywhere”* (Focus Group H).

The waste that is scattered by the wind or animals is neither swept nor removed by the waste collectors. This makes it accessible to children and waste scavengers that are at risk of being exposed to injuries and disease-causing organisms in the waste. CHWs also complained that the uncollected waste created extra work for them because they had to intervene so that their work is not undermined.

*“It is too much work for us…even the waste collectors they leave it [the uncollected waste] like that because they only collect the waste that is packaged; it is not their duty to clean up… so we have to clean it…” *(Focus Group K).

Our observations showed that irregular collection of waste made community members resort to unfriendly environmental practices. For example, in peri-urban townships, households resorted to dumping the waste illegally along the road sides, forest or bush. In some rural communities, waste is buried or burnt openly in the backyards causing the production of smoke due to incomplete combustion of waste such as diapers. We observed that waste disposal facilities are far from homes in the informal settlements. As a result, some households dig shallow pits and dispose of the waste while others dump their waste on any unoccupied spaces such as paths, cliffs, roads and streams.

### Inadequate water for practicing hygiene and sanitation

Adequate water is needed for practicing hygiene such as washing patients’ clothes and linen. In informal settlements, households do not have piped water and communal toilets. Therefore, CHWs confronted challenges in washing their patients’ clothes and linen. CHWs indicated that they had to stand in long queues to have access to water to wash linen for the patients. They also complained that there is no privacy when using communal taps and that this exposes both patients and CHWs to stigma and discrimination.

*“…When we wash the clothes everyone now knows whose clothes you are washing. You just hear them gossiping, some, pointing fingers at you...you will be waiting in the queue, they won’t give you space, they feel you will infect them…”* (Focus Group B).

Water is also needed for cleaning containers used for spiting/vomiting and buckets used for defecation by incontinent and weak patients and to flush waste water used for bed baths, vomit, urine, sputum and faeces. It is also important for cleaning the toilets and also for hand-washing after providing care to the patient. However, it was evident from observations that some households did not have piped water. Therefore, it was difficult to keep the patients clean, to flush the waste and for CHWs to wash their hands. For example, in one rural home where there was no piped water, we observed that the patient was not given a bath; a CHW was seen pouring urine in the back of the house. We also observed that the CHW neither washed the container nor her hands afterwards. Furthermore, the CHW had other patients to attend to, as a consequence, little attention was paid to the patient and little time was spent at that particular house. When asked about what we had observed, she complained about her schedule:

*“I cannot go and fetch water now, the next house where I can get water is far and I have another patient who I am supposed to take to the clinic. So I will just come back tomorrow and do everything properly…”* (CHW, community J).

#### Long distance between the house and the toilets

In most peri-urban communities, homes have flush toilets. In informal settlements, there are no flush toilets in the homes but there are flushable communal toilets located far from homes. These facilities consist of urinals and toilets with separate units for women and men. They have hand washing basins and showers and are connected to a local sewer where the effluent is channeled. The communal facilities also have storerooms and wash stands. The municipality provides all installations while the users are expected to manage the facilities. The community members appoint care takers who clean the toilets either on a voluntary basis or through a pay- peruse scheme. The care takers also liaise with the municipality on the maintenance requirements and costs. The challenge with communal toilets is that they are only open from 5 am to 6 pm daily.

The long distances between toilets and homes made CHWs reluctant to use communal toilets because they were afraid of being robbed. CHWs indicated that they had been victims of crime in the past citing instances where their belongings and the patients’ antiretroviral drugs (ARVs) were stolen from the patients’ home by the ‘Whoonga boys’ (the boys who are known to use drugs made from ARVs in the communities) while helping patients at the communal toilets. Because of this challenge, CHWs encouraged their patients to use diapers at night or to relieve themselves on the beds and they visited them on a daily basis to help change their clothes and bath them.

In rural areas, most homes have pit latrines which are located outside the house and are rarely cleaned. CHWs said that it was impossible for weak patients to walk on their own, as they needed help from family members or CHWs to access the pit latrines. Additionally, the foul odor from the pit latrines made them repulsive to use. Another challenge is that latrines were frequently vandalized by community members who stole all the roofs and doors and sold them for money to buy drugs. CHWs noted that they used bed sheets to maintain the privacy of their patients. We confirmed this assertion through observation. We also observed that because of the long distance between the homes and the communal toilets, CHWs dispose of liquid HCW like urine, waste water, sputum and vomit in the open yards. Chickens were also seen scavenging for food from such HCW.

*“… Instead of going all the way to the toilet to flush the waste water or urine or vomitus you just pour on the yard because you have other things to do…it will dry up…”* (Focus Group C).

## Discussion

We sought to understand HCWM practices by exploring the experiences of CHWs providing care to PLWHA/TB, diabetes, hypertension and other chronic illnesses. We found that the various caregiving activities carried out by CHWs in CBC generate HCW. However, some family members engaged in improper HCWM practices by refusing to help with changing diapers and removing the HCW as instructed by CHWs. This served to undermine proper HCWM practices performed by CHWs and created environmental and public health risks.

Despite receiving training on the use of personal protective equipment (PPE), some CHWs used their bare hands to handle HCW due to the shortage of gloves. This practice is contrary to the provisions of the Occupation Health and Safety Act 85 of 1993 in South Africa which states that any health care provider must not accept to handle any hazardous material without wearing PPE [41]. As the participants indicated, the inadequate supply of PPE is a consequence of the shortage of funding and material support from government and donor agencies. Research has shown that most CBOs are experiencing financial difficulties due to changes in donor agendas and the global financial crisis [21, 22]. Unfortunately this has detrimental ripple effects on HCWM.

The CHWs practice of using bare hands exposes them and the patients to the risk of infection such as HIV, hepatitis A, B and C [2, 4, 14]. Given that family members rely on CHWs for training and for modelling proper health behaviour and HCWM practices, they may emulate CHW’s improper HCWM and infection prevention and control practices. The Department of Health should work together with CBOs to ensure that CHWs are provided with adequate training on PPEs and about how to model appropriate health behaviours and practices for community members. In addition, CBOs must be provided with adequate supplies of PPE in order to motivate CHWs to engage in proper HCWM practices. The HCWM practices of HCWs should also be monitored to ensure sustainability.

The South African National Standards (SANS) on HCW management in health care facilities and other settings stipulates that all infectious waste must be segregated according to the risk it poses [40]. The Standards also specifies the method of storing and labelling different kinds of waste: infectious waste must be stored in red plastics while sharps must be stored in yellow puncture and leak proof containers labelled ‘danger, contaminated sharps’ [40]. However, we found that CHWs did not segregate HCW in homes as stipulated by the SANS. One of the factors contributing to this practice is the inadequacies in the training of CHWs. The training provided to CHWs is limited to the segregation of HCW from domestic waste and no training was provided on how to segregate HCW according to the risk it poses. The insufficient training of CHWs is reflected in the poor knowledge and skills of family members who rely on CHWs for education on how to manage HCW. Even so, none of the homes had supplies of red plastics and yellow boxes for sharps which are necessary for the segregation of waste as stipulated by the Standards [40]. The non-segregation of waste potentially exposes family members, CHWs, community members and waste handlers to physical injuries and psychological conditions like anxiety/stress disorders [2, 14]. In order to address this problem, the Department of Health should work with CBOs to train CHWs on the proper segregation of HCW. In addition, mechanisms to monitor HCW management practices by CHWs should be introduced in CBC.

Our study shows that most CBOs could not afford to supply patients with the appropriate materials and equipment such as diapers, potable urinals and bedpans meant for disposing health care waste because of a lack of funds. CHWs therefore had to improvise by encouraging patients to use buckets and tins. In addition, many of the communities had no access to potable water. Together these undermined proper hygiene practices such as keeping the patients clean, washing linen, flushing the liquid HCW, cleaning the buckets, containers and washing of hands. This could put CHWs and family members at risk of infection with bacteria that could cause diarrhoea, typhoid and leptospirosis and TB infections [2, 14, 24]. Our findings point to the need for the Department of Health, Department of Sanitation and Water and the Department of Environmental Affairs to collaborate with CBOs in order to develop interventions at the primary health care level to address issues relating to hygiene and sanitation, and HCWM at the community level. There is also a need to develop a mechanism for assisting patients in CBC with the requisite materials for disposing HCW. This could improve the well-being of the patients and prevent cross-infections in homes and the community as a whole.

The finding that most pit latrines and communal toilets were unclean, malodorous and repulsive to patients and households are similar to the findings of Roma [32] and those of Rheinlader and colleagues [42]. We found that this is a factor impeding the use of communal toilets by patients and in turn it contributes to improper HCWM practices. O’Reilly and Louis found that households are motivated to use toilets if they are comfortable, convenient, and promote privacy and dignity of an individual [43]. Previous research by Suchitra and colleagues has also highlighted the importance of designing toilets that are socially and culturally appropriate in order to promote sanitation and hygiene practices by individuals [44]. Our study calls for the South African government to provide toilet facilities that are socially acceptable and designs that are culturally appropriate in low resource communities. To ensure sustainability of such facilities, the government must involve community members in the design phase.

The irregular collection of waste by waste collectors is a major impediment to HCWM because it could expose the community and especially children to risks of infection and injuries. This reflects a larger problem of poor service delivery by the municipalities in South Africa [45, 46] which is beyond the scope of this study. This problem has been attributed to the shortage of skilled personnel to assist municipalities with rendering quality services to the people, lack of transparency in the provision of services for the people, and insufficient funds at the municipal level [46]. Research exploring the perspectives stakeholders and policy makers in HCWM in CBC could help shed more light on this issue. This is the theme that we explore in a related article which is still under development.

## Conclusions

Our findings highlight the need to incorporate the development of community health worker capacity on HCWM into CHW’s training programmes in the current primary health care initiative in South Africa. In addition, both PHC and HCWM policies should address the infrastructure deficits relating to HCWM in low resource communities. The findings have broader implications for national community health worker programmes in LMICs. In order for LMICs to develop effective community health worker programmes, there is a need for synergies in PHC and HCWM policies.

## Abbreviations

AIDS: Acquired immune deficiency syndrome
ART: Antiretroviral treatment
CBC: Community-based care
CBOs: Community-based care organisations
CHWs: Community health workers
FGDs: Focus Group Discussions
HCW: Health care waste
HCWM: Health care waste management
HIV: Human immunodeficiency virus
KZN: KwaZulu-Natal
LMICs: Low-and-middle-income countries
NHI: National health insurance
PHC: Primary health care
PLWHA: People living with HIV and AIDS
PPE: Personal protective equipment
SANS: South African national standards
TB: Tuberculosis
WHO: World Health Organization
